# Observing astrocyte polarization in brains from mouse chronically infected with *Toxoplasma gondii*

**DOI:** 10.1038/s41598-024-60304-2

**Published:** 2024-05-07

**Authors:** Yong Yao, Yaping Yuan, Shuyan Sheng, Yifan Li, Xiaoniu Tang, Hao Gu

**Affiliations:** 1 Department of Immunology, School of Basic Medical Sciences, Anhui Medical University, Hefei, China; 2 College of Life Sciences, Anhui Medical University, Hefei, 230032 China; 3 Department of Medicine, Anhui College of Traditional Chinese Medicine, Wuhu, 241002 Anhui China; 4 First Clinical Medical College of Anhui Medical University, Hefei, China; 5 School of Basic Medical Sciences, Wannan Medical College, Wuhu, 241002 Anhui China

**Keywords:** *Toxoplasma gondii*, Astrocyte polarization, Chronic infection, Microbiology, Parasitology, Pathogens

## Abstract

*Toxoplasma gondii* (*T. gondii*) is a protozoan parasite that infects approximately one-third of the global human population, often leading to chronic infection. While acute *T. gondii* infection can cause neural damage in the central nervous system and result in toxoplasmic encephalitis, the consequences of *T. gondii* chronic infection (TCI) are generally asymptomatic. However, emerging evidence suggests that TCI may be linked to behavioral changes or mental disorders in hosts. Astrocyte polarization, particularly the A1 subtype associated with neuronal apoptosis, has been identified in various neurodegenerative diseases. Nevertheless, the role of astrocyte polarization in TCI still needs to be better understood. This study aimed to establish a mouse model of chronic TCI and examine the transcription and expression levels of glial fibrillary acidic protein (GFAP), C3, C1q, IL-1α, and TNF-α in the brain tissues of the mice. Quantitative real-time PCR (qRT-PCR), enzyme-linked immunosorbent assay, and Western blotting were employed to assess these levels. Additionally, the expression level of the A1 astrocyte-specific marker C3 was evaluated using indirect fluorescent assay (IFA). In mice with TCI, the transcriptional and expression levels of the inflammatory factors C1q, IL-1α, and TNF-α followed an up-down-up pattern, although they remained elevated compared to the control group. These findings suggest a potential association between astrocyte polarization towards the A1 subtype and synchronized changes in these three inflammatory mediators. Furthermore, immunofluorescence assay (IFA) revealed a significant increase in the A1 astrocytes (GFAP^+^C3^+^) proportion in TCI mice. This study provides evidence that TCI can induce astrocyte polarization, a biological process that may be influenced by changes in the levels of three inflammatory factors: C1q, IL-1α, and TNF-α. Additionally, the release of neurotoxic substances by A1 astrocytes may be associated with the development of TCI.

## Introduction

*Toxoplasma gondii*, an intracellular parasite that must live inside a host cell, can infect a wide range of warm-blooded animals, including humans^1,2^. It is found worldwide because it can adapt to live within the cells of many mammals, including humans^3^. An estimated one-third of the world’s population has a *T. gondii* chronic infection (TCI)^4^. The infection often causes mild symptoms in healthy people, such as swollen lymph nodes and fever^5^. However, people with weakened immune systems, such as those with AIDS, are more at risk for developing serious problems throughout the body, including toxoplasmic encephalitis (TE) and ocular toxoplasmosis^6^. TE, a major cause of death associated with *T. gondii* infection, is considered one of the most serious consequences of toxoplasmosis^7^. *T. gondii* is a neurotrophic parasite that can persist in the host brain as tissue cysts during TCI, which could damage nerve cells^8^. Previous studies have shown that *T. gondii* invasion of the central nervous system can affect thinking, behavior, and emotions and even increase the risk of developing mental illness^9–11^. Studies have shown that people with various psychiatric disorders are more likely to have antibodies to *T. gondii*^12^. Anti-*Toxoplasma* antibodies have been linked to schizophrenia^13^, depression^14^, obsessive–compulsive disorder^15^, and generalized anxiety^16^.

Astrocytes, the primary supporting cells for neurons, are activated during central nervous system infection^17^. Astrocytes can take on different forms and roles under various conditions, a phenomenon known as astrocyte polarization. This cellular polarization is influenced by high levels of C1q, TNF-α, and IL-1α released by activated microglia^18^. Astrocytes also play an important role as host cells for *T. gondii*. During the tachyzoite stage, *T. gondii* proliferates within astrocytes and changes proteomic profiles of astrocytes^19–22^. In mice with acute *T. gondii* infection, an increased number of activated neurotoxic astrocytes was observed, and *T. gondii-*secreted antigens induced astrocyte polarization towards the A1 subtype through the NFκB pathway^23^. However, limited information is available regarding the role of astrocyte polarization in TCI. In the present study, we aimed to investigate astrocyte polarization and how the proportion of A1 astrocytes changes over time in TCI.

## Materials and methods

### Cell and parasite

*T. gondii* Wh6 strain, a Chinese 1 (Atypical strain, ToxoDB#9) genotype avirulent strain predominant in China, was isolated using previously described methods^24^. In vivo, infection was conducted by maintaining cysts in the brains of chronically infected mice. To collect the cysts, the brains of infected mice were mechanically homogenized in 1 ml of sterile phosphate-buffered saline (PBS). Using a light microscope, the cysts were then counted in a 10 µl brain suspension^25^.

### Mice and infection

Given that most of the mouse genes differentially expressed in response to *T. gondii* infection are similar between males and females^26^, a total of 52 female mice (weight range: 25–30 g) were divided into five groups: control group (non-infection group, n = 20), Zero day post infection group (n = 8, mouse brains were collected immediately after oral infection with tissue cysts), 1-month group (n = 8), three-month group (n = 8), and six-month group (n = 8). All were housed in pathogen-free conditions with a 12 h light/dark cycle and free access to food and water (lights on at 07:00 and off at 19:00). Daily monitor of mice was carried out by professional veterinarians at animal facility. There was no mouse died before brain collection.

The cysts of the Wh6 strain were obtained by homogenizing brain tissue in phosphate-buffered saline (PBS). Female BALB/c mice, aged seven weeks, received an intragastric administration of 30 cysts. After reaching the corresponding infection time points, the mice in the four groups were anesthetized by 1% pentobarbital solution, and their brain tissues were collected for further experiments. Five mice from each group were chosen for experiments, and all experiments were performed independently for three times. All experimental procedures were conducted in accordance with the guidelines of the Chinese Veterinary Medicine Association and approval of the Institutional Animal Care and Use Committee of Anhui Medical University (approval no. LLSC20200873). All surgery was performed under sodium pentobarbital anesthesia, and all efforts were made to minimize suffering.

### Immunofluorescence assay (IFA)

To induce anesthesia, mice were administered 1% pentobarbital solution. After anesthesia, transcardial perfusion was performed using 20 ml of ice-cold 0.01 M PBS and 20 ml of ice-cold 4% paraformaldehyde. The brains were carefully removed and post-fixed in 4% paraformaldehyde for 12 h. The brain tissues were then dehydrated in a 30% sucrose solution in 0.01 M PBS for 48 h. These tissues were then embedded in an OCT Compound (SAKURA, USA) and sliced coronally into 10–20 µm thick sections using a cryostat microtome (CM3050S, Leica, Germany).

For immunofluorescence staining, the tissue samples were blocked first with a solution containing 5% bovine serum albumin (BSA), 0.5% Triton X-100, and 0.02% normal goat serum for 2 h at room temperature. Subsequently, the samples were incubated overnight at 4 °C with primary antibodies against GFAP (1:50 dilution, Abcam, Cat#ab4648) and C3 (1:400 dilution, Abcam, Cat#ab97462). Following this, the samples were exposed to the appropriate fluorescent secondary antibodies for the primary antibodies used for 2 h at room temperature. To visualize and quantify the fluorescence intensity of astrocytes in the mouse cerebral cortex, fluorescent images were captured using an Olympus BX53 fluorescence microscope (Olympus, Tokyo, Japan). Image analysis was performed using ImageJ software (ImageJ, National Institutes of Health, Bethesda, MD). The percentage of A1 astrocyte was calculated as [number of GFAP^+^C3^+^/ (number of GFAP^+^C3^+^ + number of GFAP^+^C3^-^)] × 100%.

### Enzyme linked immunosorbent assay (ELISA)

Approximately 100 mg of mouse brain cortex tissue were homogenized thoroughly, and the resulting homogenate was then centrifuged at 12,000 × g for 15 min at 4 °C. To determine the concentrations of tumor necrosis factor (TNF)-α (BioLegend, Cat#430,901) and interleukin (IL)-1α(Invitrogen, Cat#BMS611) in the mouse brain, commercially available kits from were used according to the manufacturer's instructions. The tissue for cytokines measurement were collected between 9:00 to 11:00 AM.

### Western blotting

Proteins extracted from mouse brains, primarily the cortex, were separated using SDS-PAGE electrophoresis. The separated proteins were transferred to a polyvinylidene fluoride (PVDF) membrane. At room temperature, the PVDF membrane was blocked with 5% bovine serum albumin (BSA) for 1 h. After the blocking step, the PVDF membrane was incubated with primary antibodies overnight at 4 °C. The following primary antibodies were used in this study: anti-GFAP (1:1000 dilution, Abcam, Cat#ab7260), anti-C3 (1:2000 dilution, Abcam, Cat#ab97462), and anti-GAPDH (1:5000 dilution, Abcam, Cat#ab8245). Following incubation with the primary antibodies, the PVDF membrane was exposed to fluorescent secondary antibodies for 1 h at room temperature. To visualize the protein bands, fluorescent images were captured using the Tacon 5200 (Biotanon, China), a fluorescence imaging system. The captured fluorescent images were subsequently analyzed using ImageJ software for quantitative analysis.

### RNA extraction and quantitative reverse transcription PCR (qRT-PCR)

According to the manufacturer’s protocol, total RNA was extracted from mouse brains (cortex) using a Trizol reagent (Tiangen Biotech, China, Cat# DP424). The integrity of the isolated RNA was not evaluated in our experiments. The concentration of the extracted RNA was quantified using a NanoDrop 2000c spectrophotometer (ThermoFisher, USA)^27–29^. One microgram of the total RNA was then reverse-transcribed into cDNA using a reverse transcription kit from TaKaRa (Japan,Cat# 639,505). Quantitative real-time PCR (qRT-PCR) was performed on a QuantStudio 6 Flex real-time PCR instrument (Applied Biosystems, USA) using SYBR Green qPCR Master Mix (ThermoFisher, USA, Cat# K0251).

To analyze the gene expression data, the data were normalized to β-tubulin levels using the 2^(− ΔΔCt) method, a relative quantification method for gene expression. This method involves comparing the Ct values of the target genes with that of β-tubulin, a reference gene, to calculate the relative expression levels. The following primers were used for the analysis of β-tubulin and C1q genes:

**Table:** 

	Primer sequence	
	Forward	Reverse
Tubulin	AGAGGGAAATCGTGCGTGAC	CCAAGAAGGAAGGCTGGAAA
C1q	TCTGCCCAAGTGGCATGAG	GGAAAGGGGTGGTATAGGTCA

### Statistical analysis

All data were five values representing three independent experiments with the same conditions. Image gray values were measured using ImageJ software (version 2.1.0, National Institutes of Health, Bethesda, MD, USA). Using one-way ANOVA, between-group data were analyzed using SPSS 20.0 software (IBM Corp., Armonk, NY, USA). A p-value of less than 0.05 was considered statistically significant for all analyses. GraphPad Prism 9.0 (GraphPad Software, San Diego, CA, USA) was used for image production, and the data are presented as mean ± standard deviation (SD).

### Ethical approval

All animal experiments in this study have been approved by the Laboratory Animal Ethics Committee of Anhui Medical University (approval no. LLSC20200873) and all methods were performed in accordance with relevant regulations and guidelines including the ARRIVE guideline.

## Results and discussion

**Figure 1:**
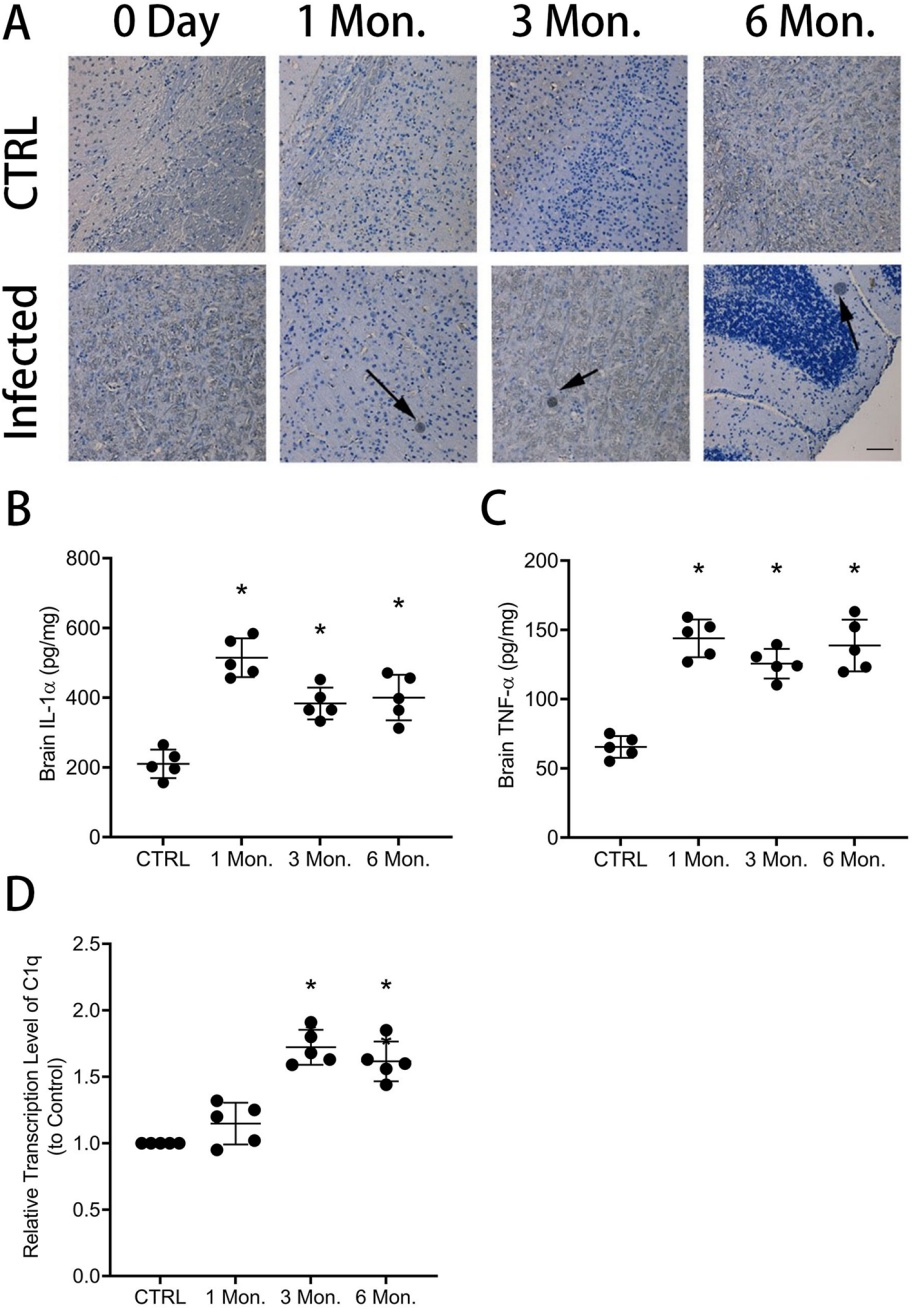
Detection of tissues cysts in brain tissue from mice with TCI (A) and evaluation of concentrations of IL-1α (B) and TNF-α (C) and transcription levels of C1q (D) in mice. The result represents the mean ± SD (n = 5), and all experiments were performed independently for three times. Bar, 50 μm. *P < 0.05, versus CTRL.

BALB/c mice were orally infected with Wh6 tissue cysts to establish a mouse model of TCI. One month post-infection, the mice in the infection group had round cysts with complete cyst walls, and a large number of bradyzoites were observed under the HE staining microscope (Fig. 1A), indicating that the model of mice chronically infected with *T. gondii* was successful.

A previous study has shown that C1q, IL-1α, and TNF-α, secreted by activated microglia, play a role in astrocyte polarization^18^. Chronic infection with an atypical *T. gondii* strain can lead to changes in the microglia population and altered behavior in mice^30^. Our study assessed the concentrations of IL-1α and TNF-α in the mouse brain by using ELISA. As depicted in Fig. 1B,C, the concentrations of TNF-α (CTRL vs. 1 Mon. vs. 3 Mon. vs. 6 Mon.: 65.52 ± 7.84 vs. 143.9 ± 13.63 vs. 125.6 ± 10.71 vs. 138.8 ± 18.69, *P* < 0.05) and IL-1α (CTRL vs. 1 Mon. vs. 3 Mon. vs. 6 Mon.: 210.3 ± 40.72 vs. 514.6 ± 55.80 vs. 383.4 ± 45.49 vs. 400.4 ± 65.45, *P* < 0.05) were increased in mice with TCI. However, compared to the levels observed during acute *T. gondii* infection, the concentrations of TNF-α and IL-1α in the TCI group were lower, indicating a lower level of inflammation in the brains of mice with chronic *T. gondii* infection. Furthermore, expression levels of C1q were evaluated by using qRT-PCR. As shown Fig. 1D, the transcription level of C1q (CTRL vs. 3 Mon. vs. 6 Mon.: 1.00 ± 0.00 vs. 1.74 ± 0.15 vs. 1.56 ± 0.14, *P* < 0.05) was found to be enhanced in the brain tissue of mice with TCI. These findings are consistent with previous studies demonstrating elevated levels of IFN-γ and TNF-α in the brains of mice chronically infected with the CK2 strain^31^. A previous study has demonstrated an upregulation of cerebral C1q in response to latent *T. gondii* infection^32^.

**Figure 2:**
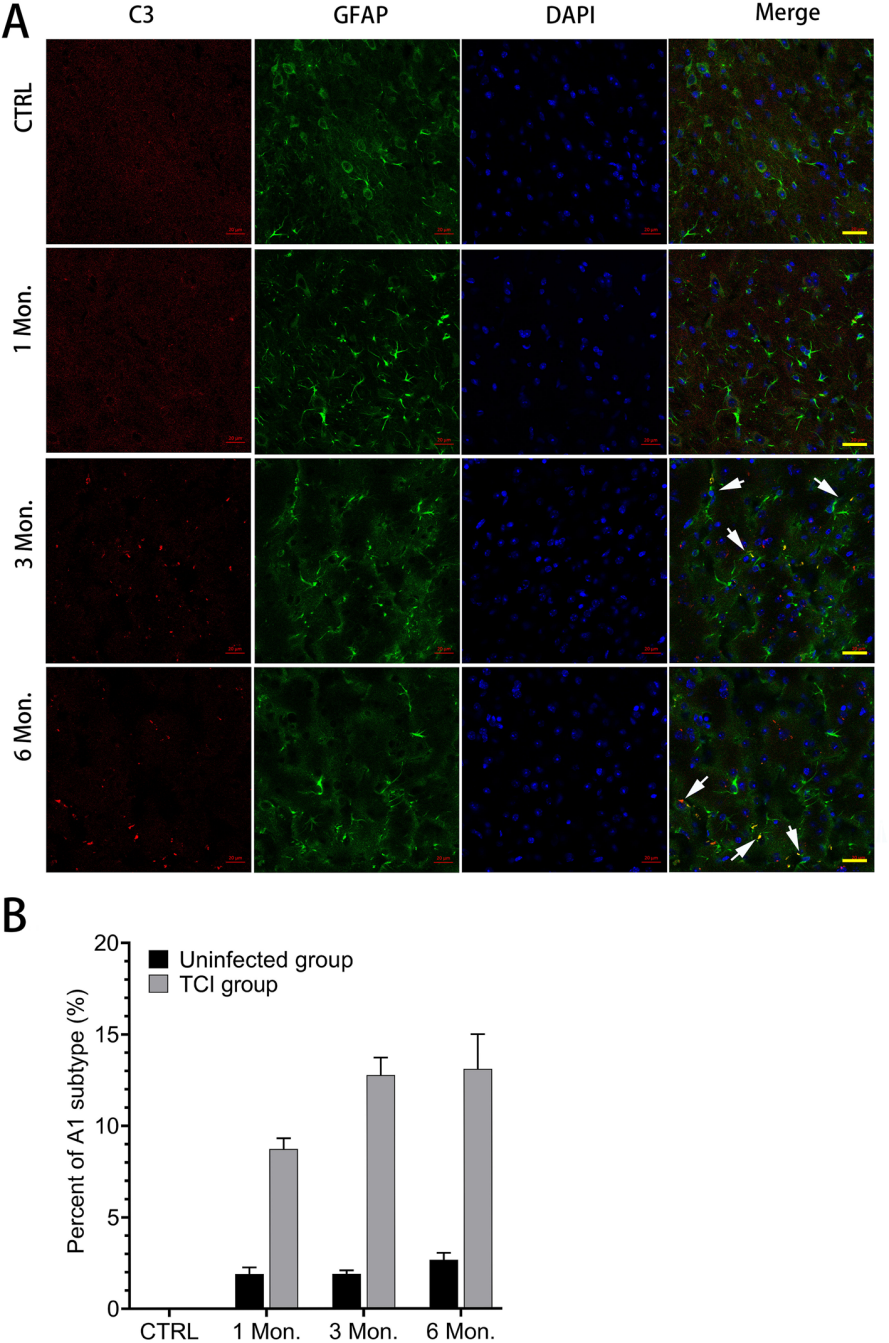
Identification of A1 astrocyte (GFAP^+^C3^+^) in the brain of mice with TCI. The percentage of A1 astrocyte was calculated as [number of GFAP^+^C3^+^/(number of GFAP^+^C3^+^ + number of GFAP^+^C3^−^)] × 100%. Each bar represents the mean ± SD (n = 5). Bar, 20 μm.

Next, the proportion of A1 astrocytes (GFAP^+^C3^+^) in mouse cortex was determined using immunofluorescence assay (IFA). As depicted in Fig. 2A,B, the proportion of A1 astrocytes (CTRL vs 1 Mon. vs 3 Mon. vs 6 Mon.: 1.24 ± 0.24 vs 7.07 ± 1.07 vs 12.59 ± 1.18 vs 13.59 ± 0.84, *P* < 0.05) was significantly higher in the TCI group compared to the control group.

**Figure 3:**
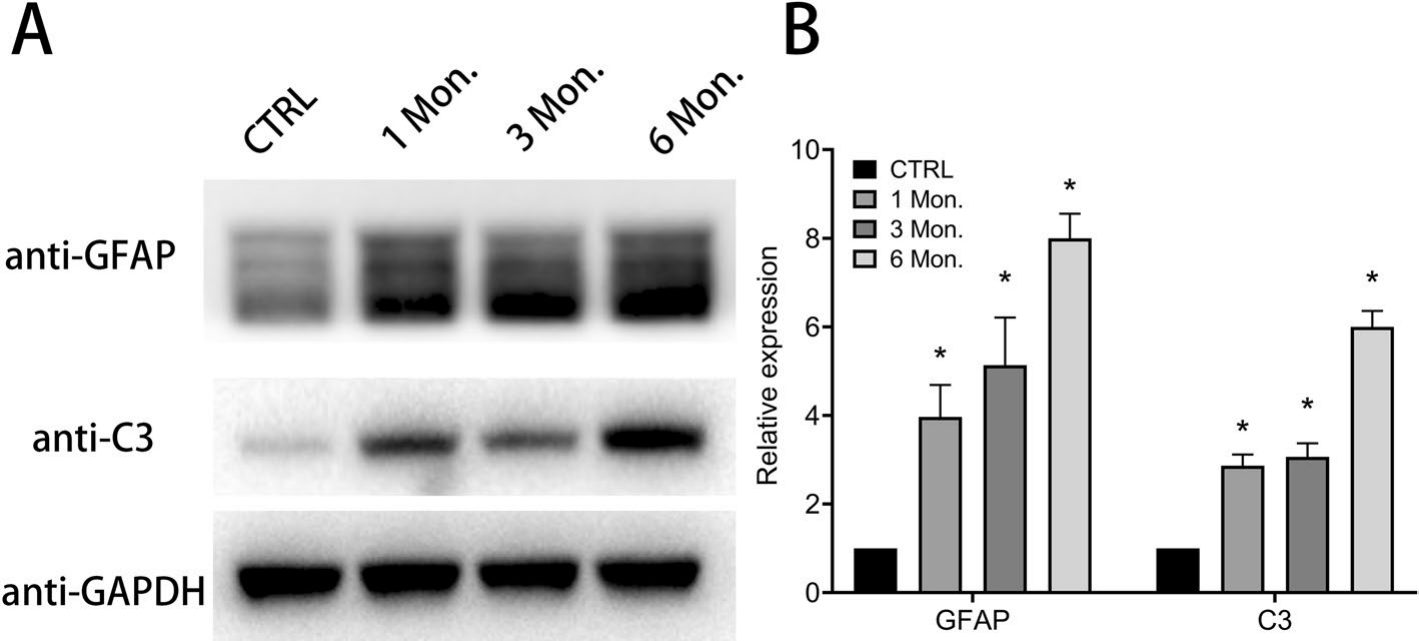
Evaluation of C3 expression level in the brain of mice with TCI. Each bar represents the mean ± SD (n = 5). *P < 0.05, versus CTRL.

Furthermore, expression levels of A1 astrocyte-specific protein C3 were detected by using western blotting. As we can see in Fig. 3A,B, higher expression levels of C3 (CTRL vs 1 Mon. vs 3 Mon. vs 6 Mon.: 1.00 ± 0.00 vs 2.87 ± 0.25 vs 3.07 ± 0.31 vs 6.00 ± 0.36, *P* < 0.05) were observed in the brains of mice in the TCI group.

The A1 phenotype is a prominent reactive astrocyte phenotype implicated in aging and neurodegenerative diseases^33,34^. In a phenotypic experiment, extracellular vesicles (EVs) derived from individuals with a reduction of deep medullary vein (r-DMVs) were observed to have the ability to disrupt the normal functions of neurons, endothelial cells, and smooth muscle cells, leading to the induction of A1 reactive astrocytes. Our present study identified the presence of A1 astrocytes in the brain tissues of mice with chronic *T. gondii* infection. A previous study demonstrated that *T. gondii* excretory-secretory antigens (TgESAs) induce astrocyte polarization towards the A1 subtype, and this polarization process can be inhibited by blocking the NFκB pathway using Bay11-7082^23^. Further investigation is warranted to identify the factors responsible for astrocyte polarization in chronic *T. gondii* infection. Additionally, exploring the correlation between astrocyte polarization and behavioral changes in animals with chronic TCI is crucial.

Based upon data presented in this study evidence indicates that chronic *T. gondii* infection (TCI) induces A1 astrocyte polarization. Consistent with previous study showing that astrocyte polarization is mediated by inflammatory factors produced by microglia^18^, TCI-induce astrocyte polarization was accompanied with changes in C1q, IL-1α, and TNF-α levels in mice brain. Saturated lipids contained in APOE and APOJ lipoparticles mediate A1 astrocyte-induced toxicity to oligodendrocytes^35^. Studies used *T. gondii* PRU strain demonstrated that from 7 days post-infection to 21 days post-infection, metabolites in the unsaturated fatty acid biosynthesis pathway in mouse cerebral cortex were upregulated as the infection progressed, indicating that *T. gondii* induces the biosynthesis of unsaturated fatty acids to promote its own growth and survival^36,37^. Thus, it is worthy of further study the linkage between TCI, host lipid metabolism and neuron damage/disfunction.

Acute infection of *T. gondii*, but not TCI, caused neuronal apoptosis in mouse brain, characterized by decreased expression level of Neu-N^8,23^. A1 astrocytes could lead to neuron death via apoptosis or ferroptosis^18,38^. The degree of negative effect of TCI on neuron’s integrity/function is worthy of detailed investigation.

The distribution of *T. gondii* genotypes varies in geographic regions. In North America and Europe, T. gondii has three main clonal lineages that are designated types I, II, and III^39^. Genotype Chinese 1 (ToxoDB#9) is dominantly circulating in mainland China^40^. Genome sequencing analysis showed that Wh6 strain of Chinese 1used in present study shared polymorphic GRA15II and ROPI/III with type I, II, and III strains^41^, indicating that Wh6 may have unique pathogenesis. Further study is needed to investigate the differences of astrocyte polarization caused by Wh6 and other tissue cyst-forming strains (Pru strain or Me49 strain). During acute *T. gondii* infection, female mice show reduced survival rates and lower cytokine levels in comparison to male mice^42^. Treatment with sex hormones, like estradiol and estrogen, increase the number of tissue cysts in brain of both male and female mice^43,44^. Transcriptional analysis showed that during chronic infection with Me49 strain, most of the host responses are similar between sexes of CBA/J mice, and females have far fewer genes that are significantly less abundant. In present study, we used female mice to establish TCI model, and in the future sex-matched experiments should be performed to investigate the different patterns of astrocyte polarization between sexes.

Although our current study has several limitations described above, we would like to provide a few directions to investigate the interaction between TCI and host neuronal pathological damage. Further research is needed to elucidate these mechanisms, investigate the functional consequences of astrocyte polarization in TCI, and assess the long-term effects of TCI on astrocyte polarization and its potential reversibility. It will also explore the functional consequences of astrocyte polarization in TCI, such as its effects on neuronal survival, synaptic function, and neuroinflammation, to identify potential therapeutic targets for mitigating the neurotoxic effects of TCI. Other potential factors involved in astrocyte polarization in TCI, beyond the inflammatory factors C1q, IL-1α, and TNF-α, will be explored to better understand the underlying mechanisms.

### Supplementary Information

Supplementary Information.

## Data Availability

The original contributions presented in the study are included in the article/Supplementary material; further inquiries can be directed to the author, Yong Yao: yaoyong@ahmu.edu.cn.
