# Supplementary material for: Observing astrocyte polarization in brains from mouse chronically infected with Toxoplasma gondii
Source: Sci Rep. 2024 May 7;14:10433. doi: 10.1038/s41598-024-60304-2 (PMC11076485; doi:10.1038/s41598-024-60304-2)

Anti-c3-01

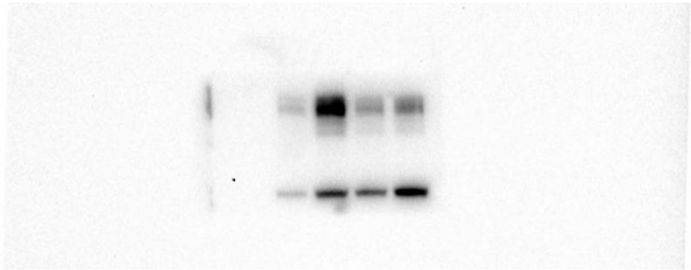

After brightness/contrast adjustment

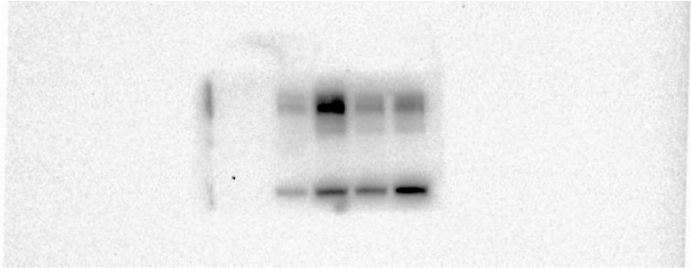

Anti-GAPDF-01

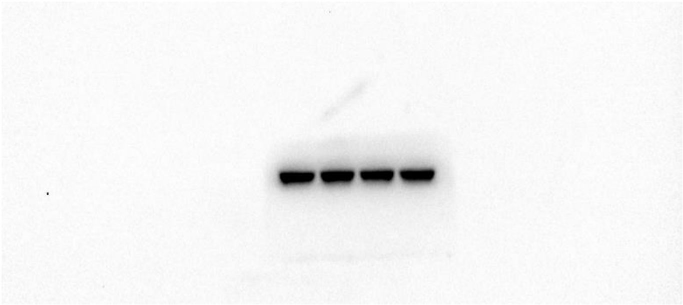

After brightness/contrast adjustment

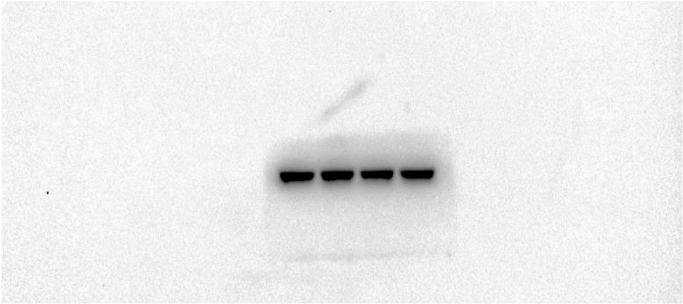

Anti-GFAP-01

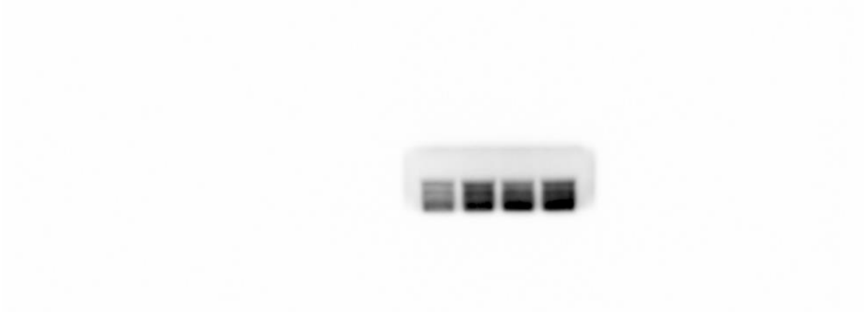

After brightness/contrast adjustment

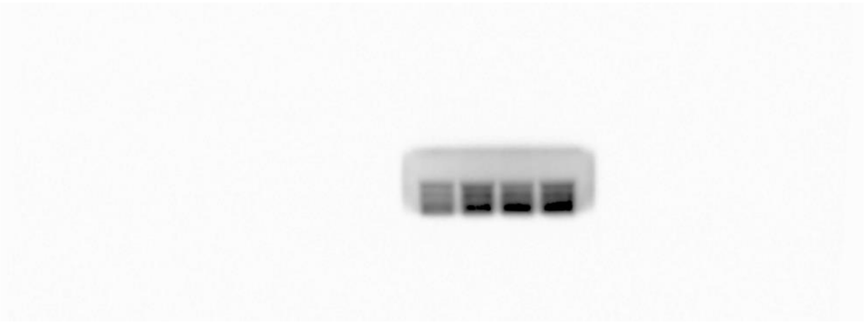

Anti-C3-02

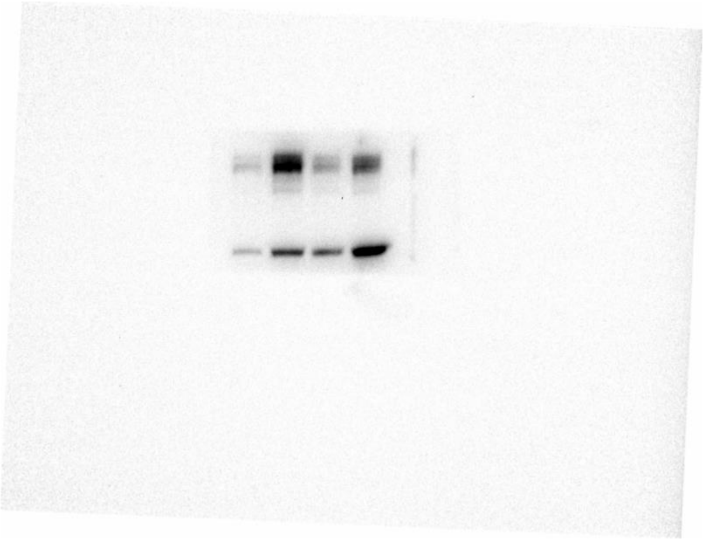

After brightness/contrast adjustment

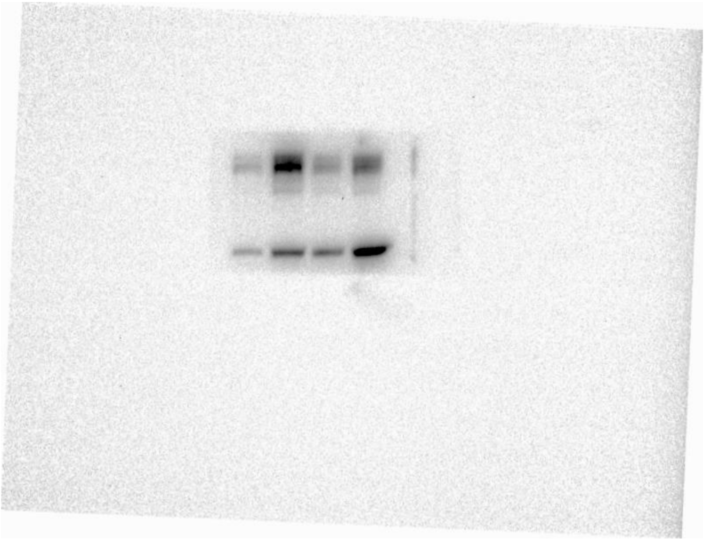

Anti-GAPDF-02

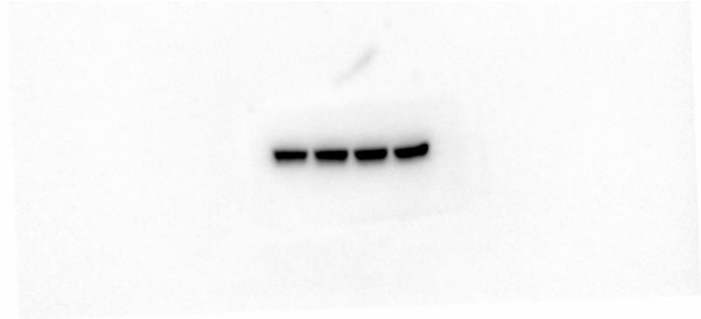

After brightness/contrast adjustment

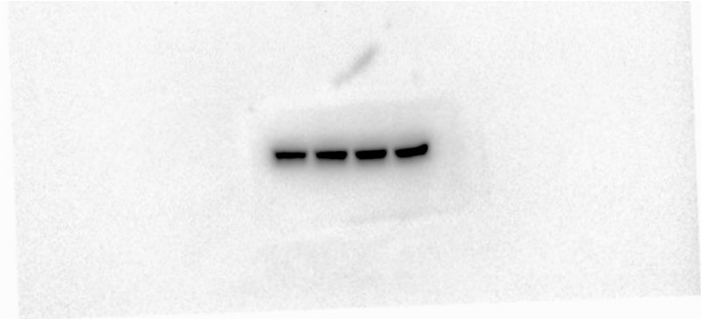

Anti-GFAP-02

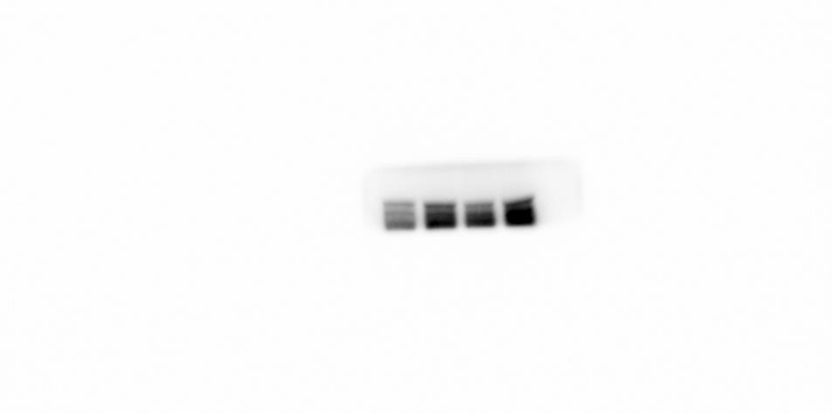

After brightness/contrast adjustment

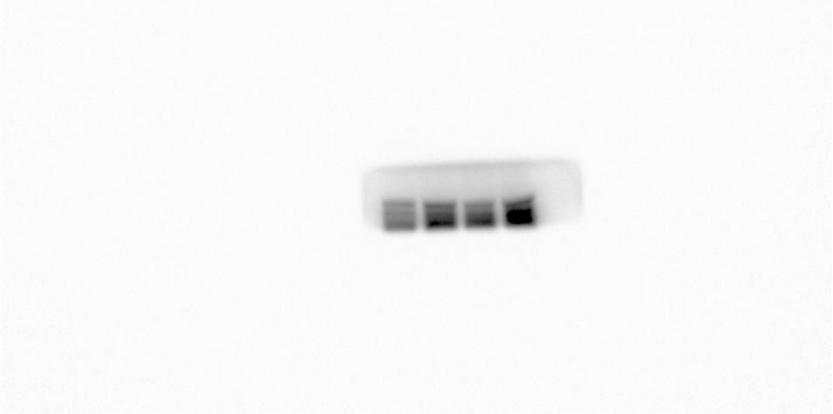

Anti-c3-03

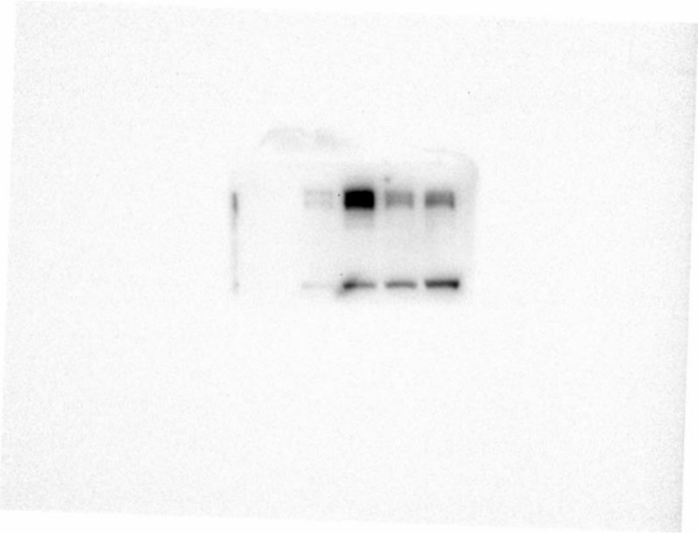

After brightness/contrast adjustment

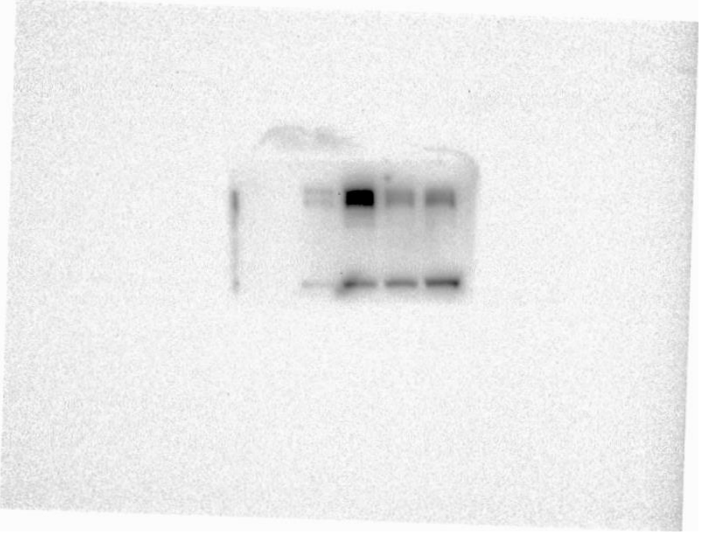

Anti-GAPDF-03

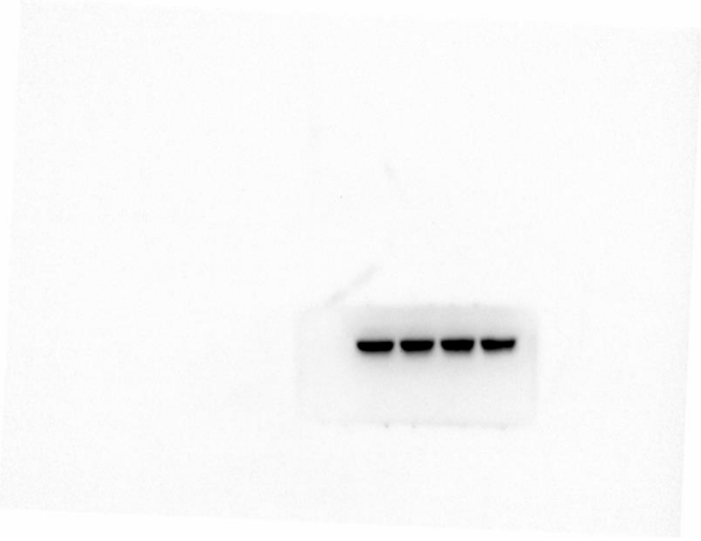

After brightness/contrast adjustment

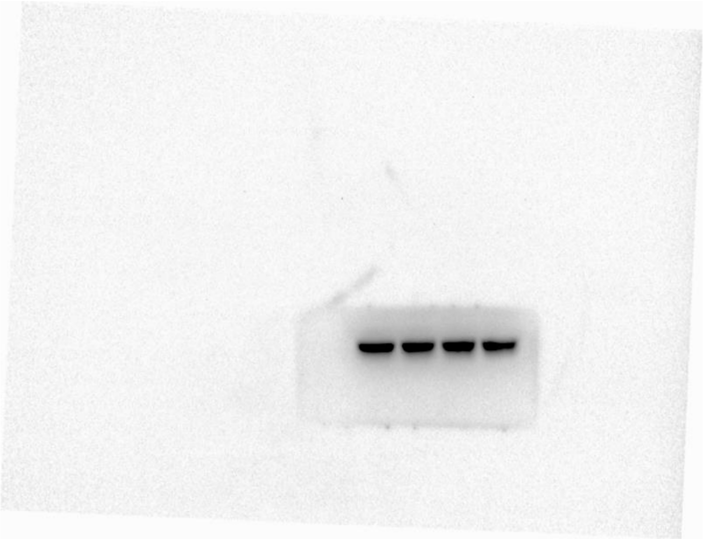

Anti-GFAP-03

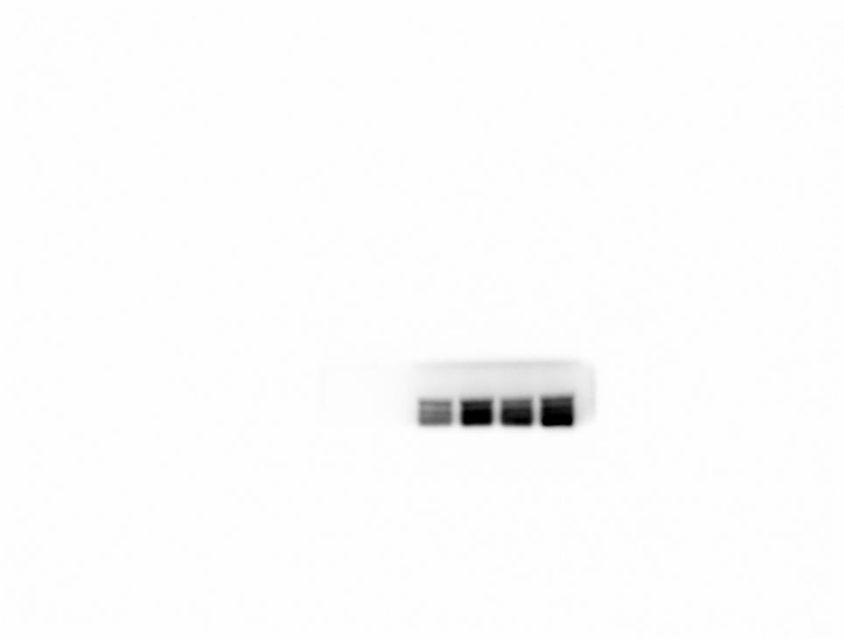

After brightness/contrast adjustment

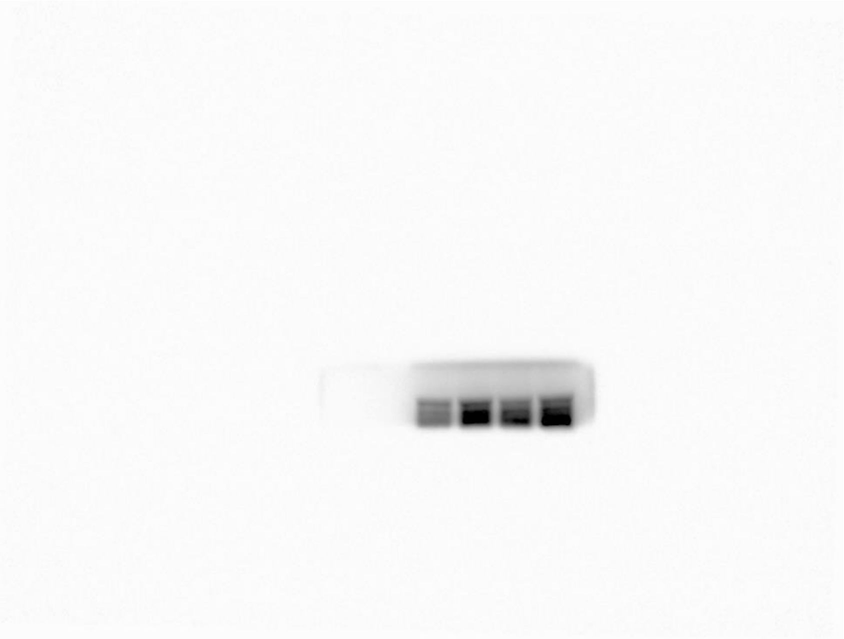

Supplement: Supplementary file 1 — Supplementary Information. [file 41598_2024_60304_MOESM1_ESM.pdf]
